# The effects of heterogeneous mechanical properties on the response of a ductile material

**DOI:** 10.1038/s41598-021-97495-x

**Published:** 2021-09-13

**Authors:** Yichi Song, Andreas Schiffer, Vito L. Tagarielli

**Affiliations:** 1 Department of Aeronautical Engineering, Imperial College London, Imperial College Road, London, SW7 2AZ UK; 2 Department of Mechanical Engineering, Khalifa University of Science and Technology, Abu Dhabi, 127788 UAE

**Keywords:** Theory and computation, Mechanical engineering

## Abstract

We investigate numerically the small-strain, elastic–plastic response of statistically isotropic materials with non-uniform spatial distributions of mechanical properties. The numerical predictions are compared to simple bounds derived analytically. We explore systematically the effects of heterogeneity on the macroscopic stiffness, strength, asymmetry, stability and size dependence. Monte Carlo analyses of the response of statistical volume elements are conducted at different strain triaxiality using computational homogenisation, and allow exploring the macroscopic yield behaviour of the heterogeneous material. We illustrate quantitatively how the pressure-sensitivity of the yield surface of the solid increases with heterogeneity in the elastic response. We use the simple analytical models developed here to derive an approximate scaling law linking the fatigue endurance threshold of metallic alloys to their stiffness, yield strength and tensile strength.

## Introduction

Numerical simulations of the response of solid materials are typically conducted assuming spatially uniform mechanical properties, with values determined by comparison to measurements. While this is a practical and pragmatic solution, which is effective in many cases, it may become inaccurate when simulating small components or materials consisting of aggregates of coarse architecture. When, on the contrary, the component analysed is substantially larger than the largest geometric features in its microstructure, it is generally assumed that modelling the spatial variability of the mechanical properties is not necessary, and that “average” or “effective” macroscopic properties can be used in analyses, assuming spatially uniform properties of the component of interest. To learn if this is really the case, in this paper we explore systematically the effects of microscopic spatial variability of materials properties on the macroscopic mechanical response of a “model” heterogeneous solid, consisting of a regular array of statistical volume elements (SVEs) possessing dissimilar, uncorrelated elastic–plastic properties. The model heterogeneous material analysed here is not intended to quantitatively represent any specific solid, but rather to embody a solid with easily tuneable heterogeneity. In a way however, we aim at representing all (isotropic) ductile solids.

Several researchers have developed multiscale modelling techniques (see for example the excellent review in^1^) to understand the links between the complexity of material microstructures and their effective, macroscopic properties. The logic followed in such models is to analyse in detail a small, representative volume element (RVE) of the material of interest and to deduce from this, via computational homogenisation, the macroscopic properties of a body. An RVE is, by definition, sufficiently large to display negligible scatter of the response, in the sense that different realisations of an RVE display approximately equal responses. This approach is sensible in absence of softening responses and strain localisation; it is nevertheless problematic, because almost all real materials consist of various solid phases, whose microscopic mechanical properties and constitutive relations are difficult (or impossible) to measure. Complex and often somewhat crude inverse problems must be solved for such models to be predictive to some extent.

An alternative, more pragmatic approach to modelling the response of solid materials while keeping into account their heterogeneity, which will also be followed in this paper, is to embrace statistical descriptions of the spatial distribution of relevant mechanical properties, as recognised by several authors^2–5^. If volume elements smaller than an RVE are considered, these will possess intrinsic variability of their mechanical response and are referred to as statistical volume elements (SVEs). Modelling a component as an array of SVEs introduces a realistic length-scale in the model, enabling realistic responses (e.g. localisation, size dependence) that cannot be captured by assuming uniform mechanical properties.

The literature on the mechanical response of heterogeneous materials is vast. Theoretical work has been conducted to determine bounds to the effective properties of heterogeneous materials and their constitutive relations^6–8^, however most of the work consists of numerical approaches. Considerable research focused on the concepts of RVE, studying its existence^9^ and its optimal size in elastic solids^10,11^, granular media^12^, elastic–plastic materials^3,13^. The existence of bounds on the response of SVEs^14,15^ and uncertainty in such response^16^ have also received substantial attention. The vast majority of the existing studies have focused on elastic solids, and those accounting for non-linear material behaviour studied two-dimensional composites. Systematic studies quantifying the effects of heterogeneity on the macroscopic material’s response are currently lacking.

In this study we will focus on 3-dimensional heterogeneous solids. We will not attempt modelling explicitly the geometry of material microstructures, but represent the microstructural heterogeneity coarsely, via arrays of SVEs. The objective is to understand the effects of the intrinsic scatter and size of such SVEs on the macroscopic mechanical response of a body. No publications have analysed in depth the multi-axial response of three-dimensional heterogeneous elastic–plastic solids comprising an array of SVEs, and we conduct such analysis here. To benchmark our numerical predictions, we also develop simple analytical bounds to the material response in uniaxial loading. The analytical models are validated and then used to propose a physically-based scaling law relating stiffness, yield strength, tensile strength and fatigue thresholds of ductile metals and alloys displaying moderate strain hardening.

We first define the problem and present the analytical models in “Analytical predictions of the uniaxial response” section, while in “Numerical calculations” we present the computational framework. Results are presented and discussed in “Results and discussion”.

## Analytical predictions of the uniaxial response

We begin by deriving approximate analytical bounds to the macroscopic uniaxial stress–strain response of elastic–plastic solids with microscopic variations of either the Young’s modulus *E*, the yield strength ${\sigma }_{y}$ or the hardening modulus *H*, defined as the slope of the true stress–strain curve in the inelastic phase; the elastic Poisson’s ratio *ν* is taken as homogeneous and equal to 0.3. We stress here that in real materials the degrees of heterogeneity in these three local properties might be correlated to some extent; here however we do not aim at representing a particular material, but rather we focus on the effects of local variations of each of these three properties separately.

We assume that the material consists of a cuboidal array of *N* elastic–plastic homogeneous cubic SVEs (or ‘cells’) with linear strain hardening; the relevant microscopic material properties are assumed to follow a uniform random distribution as follows

1 $${E}_{i}\left({r}_{i}\right)={E}_{\min}+{r}_{i}\left({E}_{\max}-{E}_{\min}\right)={E}_{\min}+{r}_{i}\Delta E$$

2 $${\sigma }_{yi}\left({r}_{i}\right)={\sigma }_{y}^{\min}+{r}_{i}\left({\sigma }_{y}^{\max}-{\sigma }_{y}^{\min}\right)={\sigma }_{y}^{\min}+{r}_{i}\Delta {\sigma }_{y}$$

3 $${H}_{i}\left({r}_{i}\right)={H}_{\min}+{r}_{i}\left({H}_{\max}-{H}_{\min}\right)={H}_{\min}+{r}_{i}\Delta H$$

with $i=1\dots N$. Here ${E}_{\max}$, ${E}_{\min}$, ${\sigma }_{y}^{\max}$, ${\sigma }_{y}^{\min}$, ${H}_{\max}$ and ${H}_{\min}$ are maximum and minimum values associated with the ranges of variation of the three mechanical properties; ${r}_{i}$ denotes uniformly distributed, uncorrelated random numbers in the interval [0,1]; the average values of the mechanical properties are denoted as ${E}_{0}$, ${\sigma }_{y0}$ and ${H}_{0}$. The choice of an uncorrelated, uniform probability density for the mechanical properties of each phase of the composite was based on the ease of implementation, on the simplicity of its analytical treatment, and the fact that it ensures the maximum possible variance for properties defined in a finite interval, such to enhance the effects of heterogeneity investigated here; we will show below that predictions are approximately insensitive to the shape of the (symmetric) probability density function considered but only to its variance.

The expected value of an upper bound for the macroscopic stress–strain response of the heterogeneous solid can be obtained assuming equal strain in all cells (Voigt bound) while an expected value of a lower bound can be calculated assuming equal stress in all cells (Reuss bound). In the following we will assume for simplicity that all cells are in a state of uniaxial stress during deformation.

### Heterogeneity in Young’s modulus

First we consider the case where the Young’s modulus *E* (stiffness of the SVEs) varies randomly across the solid according to Eq. (1), while the yield strength and hardening modulus remain uniform, i.e., $\Delta {\sigma }_{y}=0$ and $\Delta H=0$. We also assume ${H}_{0}=0$, i.e., all cells are perfectly plastic.

#### Expected value of upper bound

**Figure 1:**
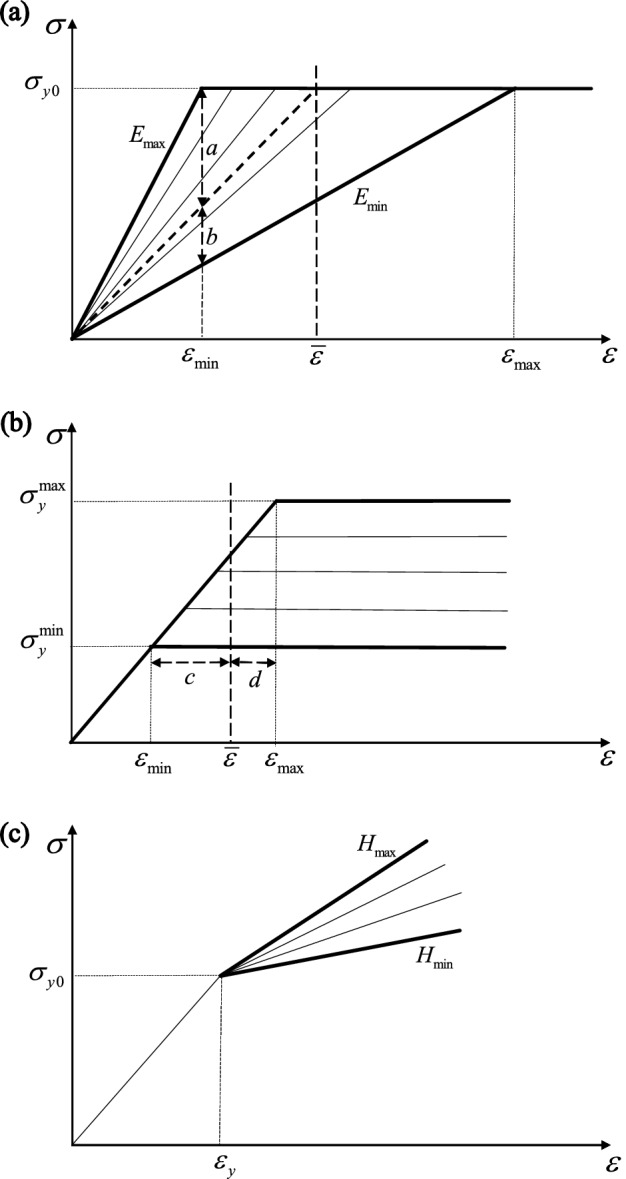
Local stress versus strain responses of the cells, for the cases of variation of (**a**) Young’s modulus, (**b**) yield stress and (**c**) hardening modulus.

The stress–strain curves of each cell of the statistical mesh are sketched in Fig. 1a; it is assumed that all cells undergo the same uniaxial microscopic strain, equal to the current macroscopic strain $\overline{\varepsilon }$. For $\overline{\varepsilon }<{\varepsilon }_{\min}$, where ${\varepsilon }_{\min}={\sigma }_{y0}/{E}_{\max}$, all cells respond elastically; if ${\varepsilon }_{\min}\le \overline{\varepsilon }\le {\varepsilon }_{\max}$, where ${\varepsilon }_{\max}={\sigma }_{y0}/{E}_{\min}$, some of the cells respond elastically while others undergo plasticity; all cells deform plastically if $\overline{\varepsilon }>{\varepsilon }_{\max}$.

For a given macroscopic applied strain $\overline{\varepsilon }$, a number *N*_p_ of cells is in the plastic regime; we define the fraction of plastic cells as

4 $${P}_{p}={N}_{p}/N$$

The fraction of cells being in the elastic regime is thus given by

5 $${P}_{e}={N}_{e}/N=1-{P}_{p}$$

where *N*_e_ is the number of elastically deforming cells.

If $\overline{\varepsilon }<{\varepsilon }_{\min}$, all cells undergo a purely elastic response, ${P}_{p}=0$. If $\overline{\varepsilon }>{\varepsilon }_{\max}$, ${P}_{p}=1$. In the transition region, ${\varepsilon }_{\min}\le \overline{\varepsilon }\le {\varepsilon }_{\max}$, with reference to Fig. 1a, the fraction of plastic cells is given by

6 $${P}_{p}=\frac{a}{a+b}=\left(1-\frac{{\varepsilon }_{\min}}{\overline{\varepsilon }}\right)/\left(1-\frac{{\varepsilon }_{\min}}{{\varepsilon }_{\max}}\right)$$

where the variables *a* and *b* represent the length of the two segments sketched in the figure. In this phase, plastic and elastic regions coexist. The homogenized (or macroscopic) stress $\bar{\sigma}$ is obtained by averaging the stresses in all cells, as follows

7 $$\bar{\sigma}\left(\overline{\varepsilon }\right)=\frac{1}{N}\left({N}_{P}{\sigma }_{y0}+\sum_{i=1}^{{N}_{e}}{E}_{i}\left({r}_{i}\right)\overline{\varepsilon }\right).$$

Combining Eqs. (4)–(7), and recalling that ${r}_{i}$ are uniformly distributed random variables, the expected value of the homogenized stress above can be calculated as

8 $$\bar{\sigma}=\frac{1}{\Delta E}\left({E}_{\mathrm{max}}{\sigma }_{y0}-\frac{{E}_{\mathrm{min}}^{2}\overline{\varepsilon }}{2}-\frac{{\sigma }_{y0}^{2}}{2\overline{\varepsilon }}\right).$$

For $\overline{\varepsilon }<{\varepsilon }_{\mathrm{min}}$ all cells respond elastically and

9 $$\bar{\sigma}=\left(\frac{{E}_{\mathrm{min}}+{E}_{\mathrm{max}}}{2}\right)\overline{\varepsilon }={E}_{0}\overline{\varepsilon }.$$

For $$\overline{\varepsilon }>{\varepsilon }_{\mathrm{max}}$$, *P*_p_ = 1 and $$\bar{\sigma}={\sigma }_{y0}$$.

#### Expected value of lower bound

Now assume that all cells are connected in series and experience an increasing but uniform macroscopic stress $\bar{\sigma}$. If $\bar{\sigma}<{\sigma }_{y0}$, the homogenized engineering strain $\overline{\varepsilon }$ is given by

10 $$\overline{\varepsilon }\left(\bar{\sigma}\right)=\frac{1}{N}\sum_{i=1}^{N}\frac{\bar{\sigma}}{{E}_{i}}=\frac{1}{N}\sum_{i=1}^{N}\frac{\bar{\sigma}}{{E}_{\mathrm{min}}+{r}_{i}\Delta E}$$

assuming small deformations. Recalling that ${r}_{i}$ are uniformly distributed random variables, we can obtain the expected value of the lower bound as

11 $$\bar{\sigma}=\frac{\Delta E \overline{\varepsilon }}{{\mathrm{ln}}\left({E}_{\mathrm{max}}/{E}_{\mathrm{min}}\right)}$$

which defines the macroscopic elastic behaviour. The response is linear elastic for $\bar{\sigma}<{\sigma }_{y0}$; when the applied stress is equal to the yield stress, the strain is undetermined (for this perfectly plastic material) and $\bar{\sigma}={\sigma }_{y0}$.

### Heterogeneity in yield stress

Next, we consider the case of the local yield stress ${\sigma }_{y}$ varying randomly across the material domain according to Eq. (2), while Young’s modulus and hardening modulus remain uniform, i.e. $\Delta E=0$, ${H}_{0}=0$ and $\Delta H=0$.

#### Expected value of upper bound

We now assume that all cells are subject to the uniform macroscopic applied uniaxial strain $\overline{\varepsilon }$. With reference to Fig. 1b, three regions of the response can be identified; for $\overline{\varepsilon }<{\varepsilon }_{\mathrm{min}}$, where ${\varepsilon }_{\mathrm{min}}={{\sigma }_{y}^{\mathrm{min}}/E}_{0}$, all cells respond elastically; a transition region exists for ${\varepsilon }_{\mathrm{min}}\le \overline{\varepsilon }\le {\varepsilon }_{\mathrm{max}}={\sigma }_{y}^{max}/{E}_{0}$, in which a fraction of the cells undergoes plasticity; for $\overline{\varepsilon }>{\varepsilon }_{\mathrm{max}}$ all cells deform plastically. In the transition region, the fraction of plastic cells *P*_p_ can be computed as

12 $${P}_{p}=\frac{c}{c+d}=\frac{{E}_{0}\overline{\varepsilon }-{\sigma }_{y}^{\mathrm{min}}}{\Delta {\sigma }_{y}}$$

where *c* and *d* denote the length of the two segments shown in Fig. 1b. The homogenized stress $\bar{\sigma}$ is the average of the stresses in all cells

**Figure 2:**
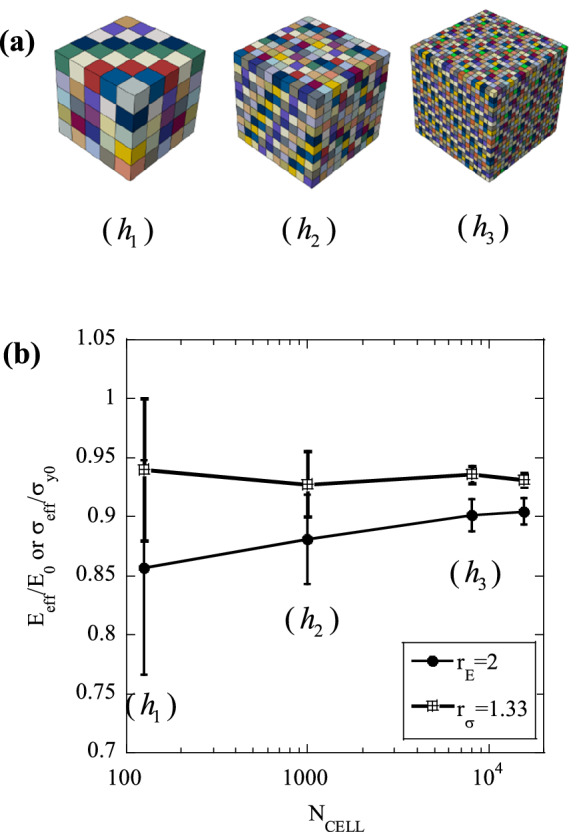
(**a**) Examples of domains with increasing ${N}_{CELL}$; (**b**) Sensitivity of the normalised effective tensile modulus, ${E}_{eff}/{E}_{0}$ (Set I) and normalised effective tensile strength, ${\sigma }_{eff}/{\sigma }_{y0}$ (Set II) to ${N}_{CELL}$.

13 $$\bar{\sigma}\left(\overline{\varepsilon }\right)=\frac{1}{N}\left({N}_{e}{E}_{0}\overline{\varepsilon }+\sum_{i=1}^{{N}_{p}}{\sigma }_{yi}\left({r}_{i}\right)\right)$$

The quantity ${\sigma }_{yi}\left({r}_{i}\right)$ in the latter equation is a uniformly distributed variable in the interval $\left[{\sigma }_{y}^{\mathrm{min}},{E}_{0}\overline{\varepsilon }\right]$. It follows that the expected value of the expression in Eq. (13) can be calculated as

14 $$\bar{\sigma}={E}_{0}\overline{\varepsilon }-\frac{ {\left({E}_{0}\overline{\varepsilon }-{\sigma }_{y}^{\mathrm{min}}\right)}^{2}}{2\Delta {\sigma }_{y}}$$

For $\overline{\varepsilon }<{\varepsilon }_{\mathrm{min}}$ the response is governed by $\bar{\sigma}={E}_{0}\overline{\varepsilon }$, while for $\overline{\varepsilon }>{\varepsilon }_{\mathrm{max}}$ the material collapses at the constant stress $\bar{\sigma}=\left({\sigma }_{y}^{\mathrm{max}}+{\sigma }_{y}^{\mathrm{min}}\right)/2={\sigma }_{y0}$.

#### Expected value of lower bound

Now assume that all cells are connected in series and subject to a uniform stress $\bar{\sigma}$. The initial response is linear elastic, $\bar{\sigma}={E}_{0}\overline{\varepsilon }$, however, as the applied stress attains the value $\bar{\sigma}={\sigma }_{y}^{\mathrm{min}}$, the material collapses at constant stress and the macroscopic strain is undefined.

### Heterogeneity in hardening modulus

Finally, we consider spatial variations of the hardening modulus *H* across the material domain according to Eq. (3), while Young’s modulus and yield stress are taken as uniform, i.e. $\Delta {\sigma }_{y}=0$ and $\Delta E=0$. In this case, all cells will yield at the same strain ${\varepsilon }_{y}={\sigma }_{y0}/{E}_{0}$, as sketched in Fig. 1c.

#### Expected value of upper bound

All cells are imposed a uniform applied strain $\overline{\varepsilon }$. In the elastic region ($\overline{\varepsilon }\le {\varepsilon }_{y}$) the macroscopic response is dictated by $\bar{\sigma}={E}_{0}\overline{\varepsilon }$, while for $\overline{\varepsilon }>{\varepsilon }_{y}$, the homogenized stress $\bar{\sigma}$ is the average of the stresses in all cells and given by

15 $$\bar{\sigma}\left(\overline{\varepsilon }\right)=\frac{1}{N}\sum_{i=1}^{N}\left({\sigma }_{y0}+\left(\overline{\varepsilon }-{\varepsilon }_{y}\right){H}_{i}\left({r}_{i}\right)\right).$$

The expected value of the expression in Eq. (15) is given by

16 $$\bar{\sigma}=\mathrm{E}\left[\bar{\sigma}\left({r}_{i}\right)\right]={\sigma }_{y0}+{H}_{0}\left(\overline{\varepsilon }-\frac{{\sigma }_{y0}}{{E}_{0}}\right).$$

#### Expected value of lower bound

Now assume that all cells are connected in series and experience the same stress $\bar{\sigma}$. For $\overline{\sigma }<{\sigma }_{y0}$ the response is elastic; for $\bar{\sigma}\ge {\sigma }_{y0}$, the global strain is given by the average of the strains in the cells according to

17 $$\overline{\varepsilon }\left(\bar{\sigma}\right)={\varepsilon }_{y}+\frac{\bar{\sigma}-{\sigma }_{y0}}{N}\sum_{i=1}^{N}\frac{1}{{H}_{\mathrm{min}}+{r}_{i}\Delta H}.$$

Similar to the analysis presented in “Heterogeneity in Young’s modulus” section, the expected value of Eq. (17) can be calculated and the homogenised stress–strain response may be written as

18 $$\bar{\sigma}={\sigma }_{y0}+\frac{\left(\overline{\varepsilon }-{\varepsilon }_{y}\right)\Delta H}{{\mathrm{ln}}\left({H}_{\mathrm{max}}/{H}_{\mathrm{min}}\right)}$$

which defines the effective strain hardening modulus as $\Delta H/{\mathrm{ln}}({H}_{max}/{H}_{min})$.

## Numerical calculations

The Monte Carlo Simulation (MCS) method was used to examine the response of elastic–plastic materials with random spatial variations of material properties under different loading conditions. The simulations were carried out in ABAQUS/Standard, conducting repeated FE simulations on 10 realisations of SVEs and evaluating the mean and spread of the populations of outputs. The details of the numerical implementation are described below.

### FE scheme

Cuboidal or prismatic SVEs of volume *V* were generated in ABAQUS/CAE via Python scripts and meshed by fully-integrated 8-noded brick elements of cuboidal shape (C3D8) and volume ${V}_{e}$. Note that geometric nonlinearity arising from large deformation were accounted for in the analyses. We define as ${N}_{FE}$ the total number of finite elements in the model ${N}_{FE}=V/{V}_{e}$. A regular tessellation was superimposed to the FE mesh to subdivide the domain into a number ${N}_{CELL}$ of cuboidal SVEs of volume ${V}_{CELL}$, such that ${N}_{CELL}=V/{V}_{CELL}$; each cell was assigned different material properties to introduce spatial inhomogeneity. The statistical tessellation was such that each cell included an integer number of equally-sized finite elements (hence ${N}_{CELL}\le {N}_{FE}$).

The elastic–plastic microscopic response of the material in each cell was modelled using linear isotropic elasticity (Young’s modulus *E*, Poisson’s ratio ν) and incompressible ${J}_{2}$ plasticity with isotropic linear strain hardening (yield strength ${\sigma }_{y}$, hardening modulus *H*). A pseudo-random number generator in Python was used to generate the values of the microscopic material properties, in accordance with Eqs. (1)–(3).

### Boundary conditions and load cases

#### Uniaxial loading in tension and compression

We performed simulations of the response in uniaxial tension and compression of prismatic materials domains, which were regularly tessellated and modelled as a set of SVEs. These simulations were performed for comparison to the analytical bounds derived in "Analytical predictions of the uniaxial response" section and to perform numerical convergence analyses. The normal displacements on a face perpendicular to the longitudinal axis of the specimen were constrained, while the opposite face was subject to normal displacements linearly increasing with simulation time, such to induce macroscopic tensile or compressive axial straining. The nodes on the lateral faces were free. Assuming uniform deformation and volume conservation during plastic deformation, the true macroscopic stresses and strains were obtained as

19 $${\sigma }_{t}={\sigma }_{n}\left(1+{\varepsilon }_{n}\right)$$

and

20 $${\varepsilon }_{t}=\mathrm{ln}\left(1+{\varepsilon }_{n}\right)$$

respectively, where the indices ‘t’ and ‘n’ denote true and nominal stresses or strains, respectively.

#### Multi-axial loading

We also simulated multi-axial loading conditions to study the effect of strain triaxiality on the response of heterogeneous cubic samples. We imposed uniform displacement boundary conditions on the faces of the cubic domain, forcing these to remain planar as deformation proceeded; as in^17^, the normal displacements of opposite lateral faces were forced to be equal and opposite; these constraints were imposed via the constraint equation tool of Abaqus, making use of appropriate auxiliary nodes. The choice of uniform over periodic boundary conditions was driven by the ease of implementation; this is expected to lead to larger size of RVEs (compared to the case of periodic boundary conditions), however this was not a problem for the simulations in this study, which involved a relatively small number of finite elements.

In this study we analysed volume elements that were initially statistically isotropic and the applied strains were kept small, such that the anisotropy induced by straining was negligible, as confirmed in preliminary checks. We therefore assumed that the material response could be evaluated in principal strain space, which coincided with principal stress space for our isotropic material. In the FE simulations we therefore prescribed time histories of only the normal macroscopic strains ${\varepsilon }_{xx}, {\varepsilon }_{yy}, {\varepsilon }_{zz}$, while the macroscopic shear strains were set to zero, ${\varepsilon }_{xy}, {\varepsilon }_{xz}, {\varepsilon }_{yz}$ = 0 (*xyz* was a reference system aligned with the edges of the cubic domain). In other words, the applied strains ${\varepsilon }_{xx}, {\varepsilon }_{yy}, {\varepsilon }_{zz}$, were interpreted as principal strains ${\varepsilon }_{I}, {\varepsilon }_{II}, {\varepsilon }_{III}$. We checked that the macroscopic shear tractions were negligibly small in the simulations, ${\tau }_{xy},{\tau }_{xz},{\tau }_{yz}\approx 0$, confirming that the global reference system *xyz* was a principal system also for the macroscopic stress tensor.

We imposed different values of strain triaxiality by subjecting the cubic domains to normal macroscopic principal strains given by

21 $$\begin{array}{l}{\varepsilon }_{1}\left(t\right)={\dot{\varepsilon }}_{0} t \left(\alpha +\beta \right)\\ {\varepsilon }_{2}\left(t\right)={\dot{\varepsilon }}_{0} t \left(-\alpha +\beta \right)\\ {\varepsilon }_{3}\left(t\right)={\dot{\varepsilon }}_{0} t \beta \end{array}$$

where *t* represents the simulation time and ${\dot{\varepsilon }}_{0}$ denotes a reference strain rate, which was set to ${\dot{\varepsilon }}_{0}=\pm 0.035 \; {\mathrm{s}}^{-1}$ in all simulations. Nine pairs of parameters $\left(\alpha ,\beta \right)$ were chosen, summarised in Table 1, to give 18 different values of strain triaxiality (defined as the ratio of the dilation to the equivalent von Mises strain) in the elastic regime, of which 9 positive (${\dot{\varepsilon }}_{0}=0.035 \; {\mathrm{s}}^{-1}$, loading cases 1–9) and 9 negative (${\dot{\varepsilon }}_{0}=-0.035 \; {\mathrm{s}}^{-1}$, loading cases 10–18). Histories of macroscopic true principal stresses and strains were extracted from each simulation from the degrees of freedom and the reaction forces of the auxiliary nodes, and used to determine the corresponding histories of hydrostatic stress ${\sigma }_{H}$ and equivalent von Mises stress ${\sigma }_{VM}$, as well as the volumetric strain ${\varepsilon }_{V}$ and equivalent von Mises strain ${\varepsilon }_{VM}$, as

**Table 1:** Loading parameters corresponding to Eq. (21).

Loading parameter	1	2	3	4	5	6	7	8	9
*α*	1	1	1	1	1	1	1	0.5	0
*β*	0	0.05	0.1	0.15	0.25	0.5	1	1	1

22 $${\sigma }_{H}=\frac{{\sigma }_{1}+{\sigma }_{2}+{\sigma }_{3}}{3}$$

23 $${\sigma }_{VM}={\left[\frac{{\left({\sigma }_{1}-{\sigma }_{2}\right)}^{2}+{\left({\sigma }_{1}-{\sigma }_{3}\right)}^{2}+{\left({\sigma }_{3}-{\sigma }_{2}\right)}^{2}}{2}\right]}^{1/2}$$

24 $${\varepsilon }_{V}\cong {\varepsilon }_{1}+{\varepsilon }_{2}+{\varepsilon }_{3}$$

25 $${\varepsilon }_{VM}=\frac{\sqrt{2}}{3}{\left[{\left({\varepsilon }_{1}-{\varepsilon }_{2}\right)}^{2}+{\left({\varepsilon }_{1}-{\varepsilon }_{3}\right)}^{2}+{\left({\varepsilon }_{3}-{\varepsilon }_{2}\right)}^{2}\right]}^{1/2}.$$

### Sensitivity of the uniaxial response to ${{\varvec{N}}}_{{\varvec{C}}{\varvec{E}}{\varvec{L}}{\varvec{L}}}$ and ${{\varvec{N}}}_{{\varvec{F}}{\varvec{E}}}$

**Figure 3:**
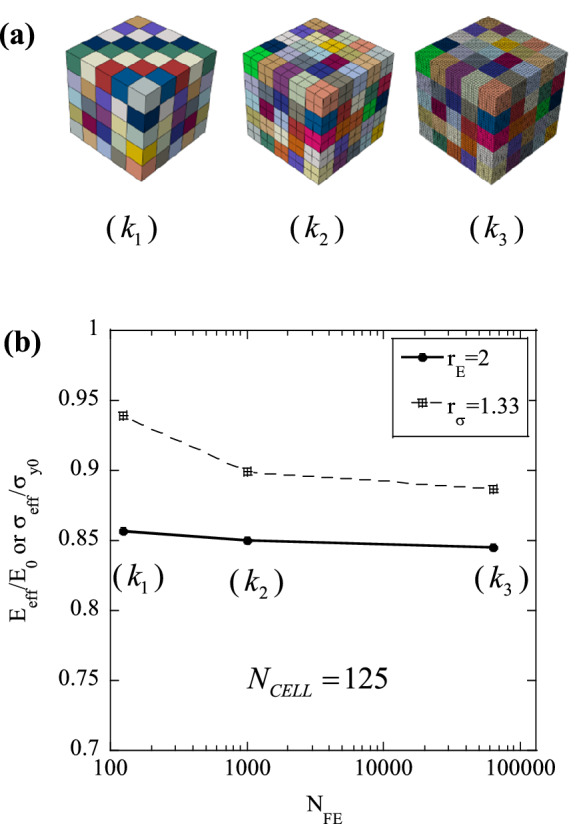
(**a**) Examples of domains with increasing ${N}_{FE}$; (**b**) Sensitivity of the normalised tensile effective modulus, ${E}_{eff}/{E}_{0}$ (Set I) and normalised tensile effective strength, ${\sigma }_{eff}/{\sigma }_{y0}$ (Set II) to ${N}_{FE}$.

**Figure 4:**
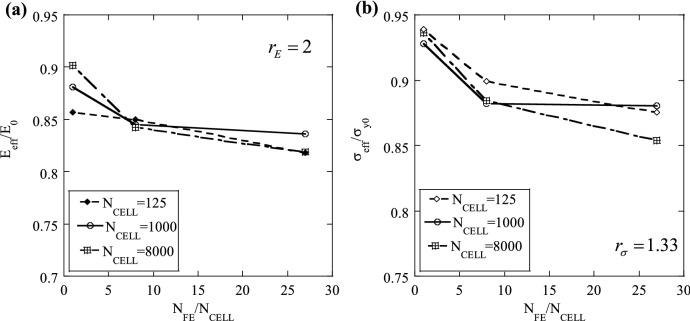
(**a**) Sensitivity of the normalised effective modulus, ${E}_{eff}/{E}_{0}$ to the number of finite elements per cell, ${N}_{FE}/{N}_{CELL}$, for Set I; (**b**) Sensitivity of the normalised effective strength, ${\sigma }_{eff}/{\sigma }_{y0}$ to the number of finite elements per cell, ${N}_{FE}/{N}_{CELL}$, for Set II.

The predictions are affected by the density of the statistical and FE meshes used, representing, respectively, a material length scale and the accuracy of the numerical discretisation. In this section we determine the sensitivity of the predictions to these parameters, with the secondary objective of determining the minimum values of ${N}_{FE}$ and ${N}_{CELL}$ which make the predictions approximately insensitive to these same parameters. Note that the results presented in this section were obtained using the loading and boundary conditions described above. Figure 2 examines the sensitivity of the predicted response to ${N}_{CELL}$, Fig. 3 focuses on the effect of ${N}_{FE}$, while Fig. 4 investigates the role of the relative fineness of the statistical and numerical discretisations, quantified by ${N}_{FE}/{N}_{CELL}$.

First, we examine the sensitivity of the predictions to ${N}_{CELL}$, for the case ${N}_{FE}={N}_{CELL}$ (i.e. each statistical cell is meshed by a single finite element) and a sample with dimensions of $5\times 5\times 5$ mm. Figure 2a shows examples of domains with ${N}_{CELL}$ varying in the range 125–8000. For each choice of ${N}_{CELL}$, MCS were performed on two sets of realisations of the microstructure. Set I was generated by implementing spatial variations of the Young’s modulus according to Eq. (1), while keeping yield strength ${\sigma }_{y0}$ and hardening modulus ${H}_{0}=0$ uniform. For Set II, random variations of the yield stress were considered, according to Eq. (2), keeping elastic modulus and hardening modulus ${(H}_{0}=0)$ uniform. We define, for Set I, a non-dimensional measure of heterogeneity ${r}_{E}$, as the relative variance of the modulus

26 $${r}_{E}=\frac{\Delta E}{{E}_{0}}$$

where Δ*E* and *E*_0_ denote range and average of the random spatial variation of Young’s modulus. Such parameter was set to ${r}_{E}=2$ (which represents an extreme case, i.e. the theoretical maximum for ${r}_{E}$) in this set of simulations. Using a similar notation, we define, for Set II, a non-dimensional measure of heterogeneity as

27 $${r}_{\sigma }=\frac{\Delta {\sigma }_{y}}{{\sigma }_{y0}}$$

A high value ${r}_{\sigma }=1.33$ was chosen in the simulations. The domains were subject to loading in uniaxial tension, which was interrupted at a total tensile strain of 0.05; the effective macroscopic modulus ${E}_{eff}$ and the 0.2% proof stress ${\sigma }_{eff}$ were extracted from the macroscopic true stress–strain histories, for both Set I and Set II, respectively.

In Fig. 2b we summarize the results of these calculations for both Set I and Set II, showing predictions of the normalised effective modulus ${E}_{eff}/{E}_{0}$ for Set I, and the normalized effective strength ${\sigma }_{eff}/{\sigma }_{y0}$ for Set II, for each choice of ${N}_{CELL}$. The error bars included in Fig. 2b represent the ranges of each output population (the distributions of outputs were found to be approximately Gaussian, as expected).

The analysis of Fig. 2b shows that ${\sigma }_{eff}/{\sigma }_{y0}$ is scarcely sensitive to ${N}_{CELL}$, but lower than the value of 1 expected in a “deterministic” simulation (in which material properties are uniformly set at their average values). The spread of such quantity decreases rapidly with increasing ${N}_{CELL}$. For Set I, the quantity ${E}_{eff}/{E}_{0}$ initially increases with ${N}_{CELL}$ but this dependence becomes small for ${N}_{CELL}>1000$. Again, the scatter in this normalised measure of strength decreases rapidly with increasing ${N}_{CELL}$. Increasing the value of ${N}_{CELL}$, while the size of the domain simulated is kept constant, is physically equivalent to increasing the size of the component analysed. Therefore our simulations suggest a size dependence of the tensile response for Set I, such that larger samples are stiffer than smaller ones, as reported by several authors (e.g.^18^).

Next, we examine the sensitivity of the FE predictions to ${N}_{FE}$, with ${N}_{CELL}$ held constant. FE simulations were performed on a single realisation of the heterogeneous solid, with ${N}_{CELL}=125$, and the FE mesh was progressively refined, as illustrated in Fig. 3a. Again, two sets of simulations were performed, one with ${r}_{E}=2$ (Set I) and the other with ${r}_{\sigma }=1.33$ (Set II). Each domain was subjected to uniaxial tension and the simulations were interrupted when a total tensile strain of 0.05 was reached.

Figure 3b presents, on the same graph, plots of the normalised effective modulus ${E}_{eff}/{E}_{0}$ for Set I (${r}_{E}=2$) and the normalized effective strength ${\sigma }_{eff}/{\sigma }_{y0}$ for Set II (${r}_{\sigma }=1.33$), for each choice of ${N}_{FE}$. It can be seen that both ${E}_{eff}/{E}_{0}$ and ${\sigma }_{eff}/{\sigma }_{y0}$ decrease monotonically with increasing ${N}_{FE}$; the decrease in stiffness is expected, as the number of degrees of freedom of the calculation increases; the decrease in strength is justified by the fact that a progressively refined FE mesh better captures the stress concentrations induced by the microscopic variations of mechanical properties.

The latter mesh sensitivity study was repeated for higher values of the statistical mesh density, namely ${N}_{CELL}=1000$ and ${N}_{CELL}=8000$, for both Set I and Set II. The predictions are presented in Fig. 4, where ${E}_{eff}/{E}_{0}$ is plotted as a function of the ratio ${N}_{FE}/{N}_{CELL}$ in Fig. 4a for Set I (${r}_{E}=2$), while for Set II (${r}_{\sigma }=1.33$), predictions of ${\sigma }_{eff}/{\sigma }_{y0}$ are shown Fig. 4b. Clearly, the choice of ${N}_{CELL}$ does not have a notable effect on the sensitivity of the predictions to ${N}_{FE}/{N}_{CELL}$; predictions are scarcely sensitive to the FE mesh if at least 8 finite elements are used to mesh each SVE (i.e. ${N}_{FE}/{N}_{CELL}=8$), irrespective of the chosen ${N}_{CELL}$.

## Results and discussion

### Uniaxial response

In this section we analyse the numerical predictions of the uniaxial response in detail and compare them to the bounds derived analytically above. We chose prismatic volume elements (VEs) of square cross-section, consisting of 20,736 cubic cells ${(N}_{CELL}={20{,}736})$ and each cell was meshed with a single finite element (${N}_{FE}={N}_{CELL}$). The prismatic domains analysed had dimensions of $6\times 4\times 4 \; \mathrm{mm}$, to represent the gauge portion of test specimens typically used for laboratory-scale mechanical tests. As shown in Fig. 2b, this choice of ${N}_{FE}, {N}_{CELL}$ makes the predictions of average modulus and strength insensitive to further refinements of the discretisation. All the numerical predictions presented in this section are average responses obtained from 10 simulations of different realisations of the heterogeneous specimens.

#### Effect of variation of Young’s modulus

**Figure 5:**
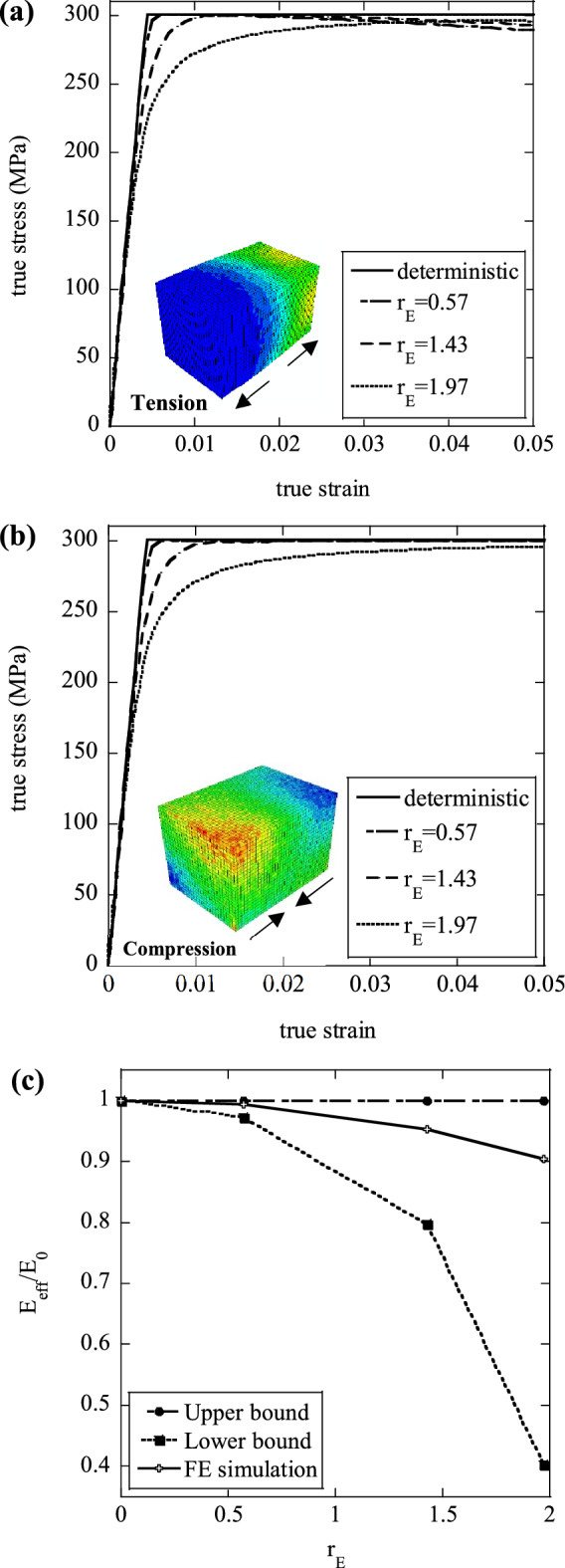
Effect of the variation of modulus on the macroscopic (**a**) tensile and (**b**) compressive stress–strain response; (**c**) Normalised effective modulus, ${E}_{eff}/{E}_{0}$ as a function of the degree of heterogeneity ${r}_{E}$; the insets in figures (**a**) and (**b**) are contour plots of the absolute value of the maximum principal strain at a true macroscopic strain of 0.05.

We now present the results of simulations in which a random spatial variation of Young’s modulus was imposed, with uniform yield strength and uniform hardening modulus, *H* = 0 (as sketched in Fig. 1a). The average macroscopic uniaxial true stress versus true strain histories are presented in Fig. 5a for the case of tension and in Fig. 5b for the case of compression. Simulations were conducted at three different values of ${r}_{E}$ as indicated; the insets show contours of the absolute value of the maximum principal strain at a 5% total macroscopic axial strain (for the maximum value of ${r}_{E}$ considered). A “deterministic” simulation, in which all material properties were taken as uniform and equal to the average values $({E}_{0}=70 \; \mathrm{GPa}; {\sigma }_{y0}=300 \; \mathrm{MPa}; H=0)$ is included in the figures for comparison.

In both tension and compression, a non-linear elastic–plastic response begins at macroscopic stress well below ${\sigma }_{y0}=300 \; \mathrm{MPa}$, as a consequence of the stress concentrations and multiaxial stress states induced by the variations of Young’s modulus; the stress at the onset of plasticity decreases with increasing ${r}_{E}$. At higher stresses, the material exhibits a non-linear macroscopic response reminiscent of strain-hardening, but non-linearity is rather a consequence of the progressive yield of more and more cells, similar to what described by Eq. (8) (we recall that the microscopic material response is perfectly plastic). The peak true stress is scarcely sensitive to the imposed variation of Young’s modulus in the ranges investigated and is always close to the homogeneous microscopic yield stress of 300 MPa; however, this decreases with the degree of heterogeneity.

At relatively large strains, the mechanisms of deformation are different in tension and compression. In tension the deformation, initially uniform, localises and a neck forms in the sample at a random location, as evident from the inset of Fig. 5a. Due to the local reduction of cross-sectional area in the neck, the macroscopic response exhibits a stress peak followed by geometric softening. Furthermore, the macroscopic strain at which a peak in tensile stress is attained is sensitive to ${r}_{E}$: the higher ${r}_{E}$, the higher the strain at peak stress; this suggests that the ductility of a heterogeneous material increases with the degree of elastic heterogeneity, as quantified in this case by the parameter ${r}_{E}$. In compression, on the other hand, the necking mechanism is absent and the macroscopic response progressively evolves towards a plateau response at stress ${\sigma }_{y0}=300 \; \mathrm{MPa}$, as shown in Fig. 5b. Some inhomogeneity in the strain field is visible, due to the tendency of the specimen to localise deformation along inclined planes. In the apparent hardening phase of both the tensile and compressive responses, the flow stress at any given macroscopic strain decreases with increasing ${r}_{E}$. Figure 5c presents a comparison between the numerically predicted normalised effective tensile modulus and the expected value of the analytical bounds developed above. It can be seen that the average values of our numerical predictions fall between the analytically predicted expected values of the upper and lower bounds.

#### Effect of variation of yield stress

**Figure 6:**
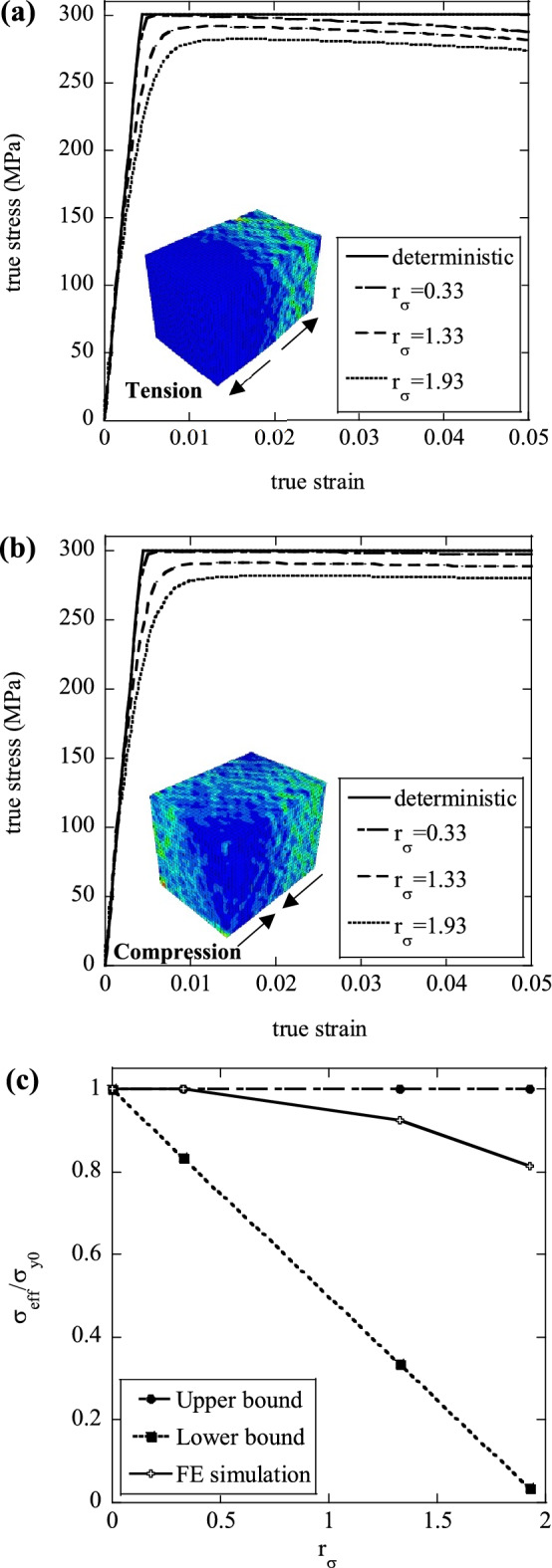
Effect of the variation of yield strength upon the macroscopic (**a**) tensile and (**b**) compressive stress–strain response; (**c**) Normalised effective strength, ${\sigma }_{eff}/{\sigma }_{y0}$, as a function of the degree of heterogeneity ${r}_{\sigma }$; the insets in figures (**a**) and (**b**) are contour plots of the absolute value of the maximum principal strain at a true macroscopic strain of 0.05.

In Fig. 6a,b we present average macroscopic true stress versus true strain histories obtained from simulations in which the yield stress was varying in space, while the Young’s modulus and hardening modulus were kept uniform $\left({E}_{0}=70 \; \mathrm{GPa}; \; {\sigma }_{y0}=300 \; \mathrm{MPa}; \; H=0\right)$. Predictions are presented for three different levels of heterogeneity, quantified by the values of ${r}_{\sigma }$ indicated. The insets show contours of the absolute value of the maximum principal strain at 5% total macroscopic axial strain.

The effects of a microscopic variation of yield stress are somewhat different from those observed for variations in Young’s modulus. In both tension and compression, compared to the deterministic simulation, the macroscopic response of the heterogeneous material features an onset of plasticity at relatively low values of stress, followed by a non-linear (apparent) strain hardening phase, consequence of the progressive yielding of the cells in the sample. Again, a higher degree of heterogeneity ${r}_{\sigma }$ results in lower flow stress at any applied macroscopic strain. In tension and compression, deformation localises along shear bands at approximately ±45° on the loading axis; in tension these bands evolve to trigger a macroscopic necking mechanism associated with strain softening, while in compression, the predicted stress quickly reaches a plateau after the initial elastic phase, as shear bands spread across the whole sample. In tension, the macroscopic strains at necking increase with ${r}_{\sigma }$, reinforcing the notion that material heterogeneity increases macroscopic ductility.

In Fig. 6c numerical predictions of the 0.2% proof stress in tension are compared to the analytical bounds. As expected, these lie within the analytical bounds but appear much closer to the upper bound than to the lower bound.

#### Effect of probability distribution

**Figure 7:**
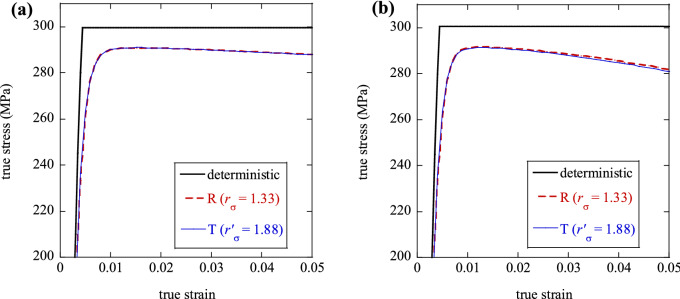
Comparison between predictions obtained with a uniform rectangular distribution (R) and a symmetric triangular distribution (T) of random yield strength with the same mean and variance: (**a**) tension and (**b**) compression.

We performed additional MCSs using a symmetric, triangular (T) probability density function of yield stress, to compare with our previous results obtained for the case of a uniform, rectangular distribution (R) with ${r}_{\sigma }=1.33$ (Fig. 6). The range of variation for the triangular probability density, ${r{^{\prime}}}_{\sigma }=\Delta {\sigma {^{\prime}}}_{y}/{\sigma {^{\prime}}}_{y0}$, was chosen such to obtain the same average and variance as for the uniform distribution with ${r}_{\sigma }=1.33$. This was achieved by setting ${r{^{\prime}}}_{\sigma }=\sqrt{2} {r}_{\sigma }=1.88$ and ${\sigma {^{\prime}}}_{y0}={\sigma }_{y0}=300 \; \mathrm{MPa}$. In Fig. 7 we compare the average macroscopic stress–strain responses obtained with the uniform distribution (${r}_{\sigma }=1.33$) to those obtained with a triangular distribution of equivalent variance (${r{^{\prime}}}_{\sigma }=1.88$). Clearly the two types of predictions are nearly coincident in both tension (Fig. 7a) and compression (Fig. 7b), suggesting a scarce sensitivity of our predictions to the assumed shape of the probability density functions which govern the spatial variation of material properties. It would be interesting to examine the case of a non-symmetric probability density function of the same average and variance, which is omitted here for brevity.

#### Effect of variation of hardening modulus

A separate set of simulations was conducted by imposing a spatial variation of the material hardening modulus, with elastic modulus and yield stress kept constant $\left({E}_{0}=70 \; \mathrm{GPa}; \; {\sigma }_{y0}=300 \; \mathrm{MPa}\right)$. The mean value of the hardening modulus was ${H}_{0}=35 \; \mathrm{Gpa}$ and the degree of variation was chosen as ${r}_{H}=2$.

The ensuing macroscopic response, not shown for the sake of brevity, comprised a linear elastic part followed by a linear strain-hardening phase. The effective hardening modulus, for the (relatively high) degree of variation ${r}_{H}$ considered, was much closer to that predicted by the upper bound developed above (Eq. (16)) than to the corresponding lower bound (Eq. (18)), indicating cooperative straining. A spatial variation of the hardening modulus alone does not have substantial effects on the macroscopic response, with the exception of resulting in a macroscopic hardening modulus only slightly smaller than the average microscopic value (as predicted by the expected value of upper bound, Eq. (16)).

#### Size (and shape) dependence of the response

Introducing a spatial variation of material properties in the numerical models introduces a length scale in the problem, namely the dimension of the SVE used. Dimensional analysis dictates that the problem must depend on a non-dimensional parameter involving this cell dimension, for example the ratio between size of the material sample and size of the statistical cells, which increases monotonically with decreasing ${N}_{CELL}$. The number of SVEs (or cells) used, ${N}_{CELL}$, is representative of physical size of the material specimen. The dependence of the macroscopic response upon ${N}_{CELL}$ has been already highlighted in Fig. 2. We now examine in detail how the macroscopic stress–strain response changes as a function of ${N}_{CELL}$.

**Figure 8:**
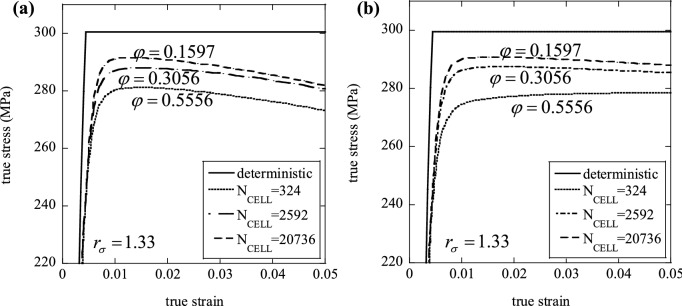
Effect of ${N}_{CELL}$ on the macroscopic (**a**) tensile and (**b**) compressive response, for a given degree of plastic heterogeneity, ${r}_{\sigma }=1.33$.

First, consider the case of uniform elastic modulus, uniform hardening modulus and non-uniform yield stress $$({r}_{\sigma }=1.33$$, $${r}_{E}={r}_{H}=0)$$. Figure 8a,b present, respectively, the tensile and compressive stress–strain curves for a heterogeneous material, compared to the deterministic (homogeneous) case. Each figure includes three curves corresponding to different values of $${N}_{CELL}$$. We include the corresponding value of the ratio $$\varphi$$, defined as the number of material cells lying on the specimen’s lateral surface divided by those lying within the specimens. The latter ratio depends on $${N}_{CELL}$$ through a simple geometric relation and it also depends on the shape of the specimen analysed. It is seen from Fig. 8 that the material displays an inverse size effect in both tension and compression, with larger specimens (or equivalently, specimens with larger values of $${N}_{CELL}$$) displaying a stronger material response. This has been reported by other studies on size effects in the response of ductile elastic–plastic solids (e.g.^19^) and is justified in terms of the different stress states of material domains lying on the surface of the solids and those contained within this surface. In fact, material cells lying with at least one side on the external surface are less constrained, having at least one vanishing principal stress, and tend to yield earlier than material cells within the bulk of the SVE. Our results support this notion, reporting a weaker response in both tension and compression for higher values of $$\varphi$$. We note that assuming a uniform material response would not give rise to such size effect, but modelling the heterogeneity in material properties results in a dependence of the macroscopic response upon both size and shape of the specimen analysed.

In the data presented in Fig. 8 the aspect ratios of the specimens analysed were kept constant. In a separate set of simulations $$({r}_{\sigma }=1.33$$, $${r}_{E}={r}_{H}=0,{N}_{FE}={N}_{CELL})$$, we varied the geometry of the SVE substantially, ranging from a chain of cells in series to an array of cells all arranged in parallel; the corresponding uniaxial tensile strength varied from the mean yield stress (for the case of a parallel arrangement, in line with the upper bound derived in “Analytical predictions of the uniaxial response” section) to approximately one third of this value, for the case of a chain arrangement. Such variations are driven by the dependence of $$\varphi$$ upon the geometry (aspect ratios) of the SVE, but also by different mechanisms of deformation and different stress triaxiality induced by the specimen geometries.

**Figure 9:**
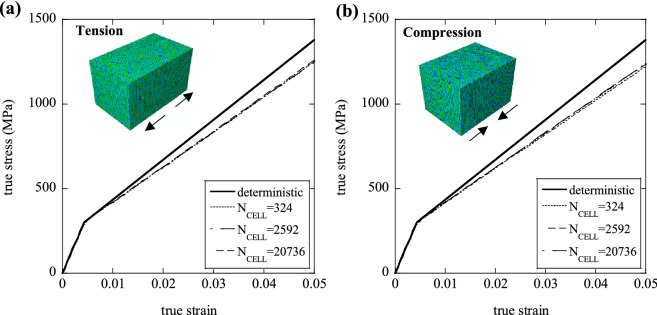
Effect of ${N}_{CELL}$ on the macroscopic (**a**) tensile and (**b**) compressive response, for a given degree of heterogeneity in the hardening modulus, ${r}_{H}=2$; the insets represent contour plots of equivalent plastic strain at a true strain of 0.05.

Next we present, in Fig. 9, analogous information as that in Fig. 8, for the case of a spatial variation of the hardening modulus alone $({r}_{H}=2$, ${r}_{E}={r}_{\sigma }=0)$; the Young’s modulus and yield strength were chosen as ${E}_{0}=70 \; \mathrm{GPa}$ and ${\sigma }_{y0}=300\; \mathrm{MPa}$, respectively, and the mean value of hardening modulus was ${H}_{0}=35 \; \mathrm{GPa}$. The observed size effect is in this case very mild in the wide range of sizes ($324\le {N}_{CELL}\le {20{,}736}$) considered here.

### Multiaxial response

**Figure 10:**
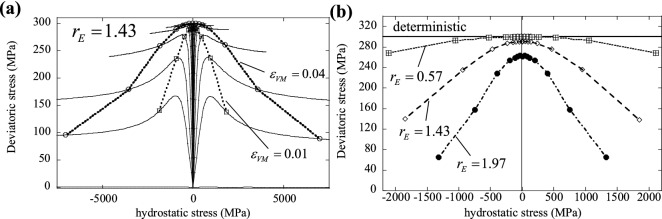
(**a**) Yield surface evolution for a given degree of elastic heterogeneity, ${r}_{E}=1.43$. (**b**) Effect of ${r}_{E}$ on the yield surface.

To illustrate the effects of material heterogeneity on the multiaxial response, we used the loading and boundary conditions described in the “Multi-axial loading” section to construct pseudo-yield surfaces of heterogeneous solids and compare with the homogeneous case. Cubic SVEs were generated with ${N}_{FE}={N}_{CELL}={21{,}952}$, using a $5\times 5\times 5\; \mathrm{mm}$ specimen. Macroscopic strain histories were imposed on the SVEs according to Eq. (19) and Table 1. Two sets of simulations were conducted, varying either the elastic modulus or the yield stress at microscopic scale. All pseudo-yield surfaces were constructed using the average responses obtained from 10 simulations of different realisations of the heterogeneous cubic domain. Figure 10 shows the yield surface of a heterogeneous material featuring microscopic variations of the elastic modulus. In Fig. 10a we present, for ${r}_{E}=1.43$, the evolution of the yield surface. Trajectories of the hydrostatic and deviatoric stresses at different (initial) stress triaxiality are shown, and on each trajectory two points are marked, corresponding to macroscopic von Mises equivalent strains of 0.01 (squares) and 0.04 (circles). Clearly the macroscopic yield surface is pressure-dependent, although the microscopic material response is pressure-insensitive (obeying the von Mises criterion); this is due to the fact that an imposed macroscopic hydrostatic strain results in deviatoric stress components at microscopic level, due to the elastic heterogeneity. The macroscopic material hardening can be deduced from the figure and is neither isotropic nor kinematic (note that isotropic hardening is imposed at microscopic level). Figure 10b shows the effect of the degree of heterogeneity ${r}_{E}$ on the multiaxial response: clearly, a higher heterogeneity gives a weaker response and enhances the pressure-sensitivity of the macroscopic response (note that the yield surfaces in Fig. 10b are obtained at macroscopic von Mises equivalent strains of 0.01).

**Figure 11:**
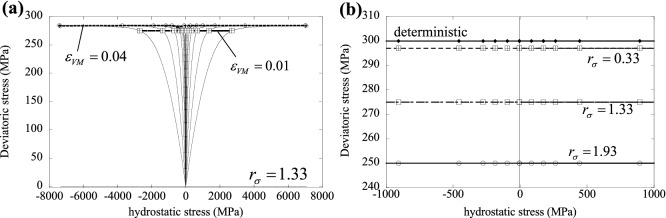
(**a**) Yield surface evolution for a given degree of plastic heterogeneity, ${r}_{\sigma }=1.33$. (**b**) Effect of ${r}_{\sigma }$ on the yield surface.

Figure 11 presents similar information for the case of microscopic variations of the yield stress, with uniform elastic modulus; Fig. 11a shows the evolution of the yield surface for ${r}_{\sigma }=1.33$, while Fig. 11b presents the effect of varying ${r}_{\sigma }$ on the macroscopic yield surface. In this case the macroscopic response is pressure-insensitive and hardening is isotropic, as for the local constitutive response; this is due to the fact that the material is elastically homogeneous and macroscopic hydrostatic stress components do not induce microscopic deviatoric components in this case. Again, a higher degree of heterogeneity results in a weaker macroscopic response (Fig. 11b).

### Can we predict the fatigue endurance limit from the quasi-static response?

The discussion of the results presented above has highlighted, among other things, the fact that in real materials the onset of yield occurs at relatively low stresses (see Figs. 5 and 6), much lower than what is commonly referred to as the material ‘yield stress’ (i.e. proof stress, or macroscopic flow stress at 0.2% plastic strain). In addition, we found that the macroscopic response of heterogeneous materials featuring variations of the yield stress is close (Fig. 5) to the predictions of the simple upper bound model developed in “Analytical predictions of the uniaxial response” (Eq. (14)). In principle, fitting Eq. (14) to the stress strain curve of a material with small macroscopic strain hardening may be used to determine the macroscopic stress at the onset of local plasticity, ${\sigma }_{y}^{\mathrm{min}}$.

Such fitting exercise was conducted using uniaxial tension data by Bettaieb et al.^20^ on two types of Titanium alloys, considering only the stress–strain histories for strains less than 0.01; an initial linear response was assumed, followed by a non-linear response (given by Eq. (14)) for $\sigma >{\sigma }_{y}^{\mathrm{min}}$. The value of ${\sigma }_{y}^{\mathrm{max}}$ was taken as the flow stress at total strain of 0.01 (this value approximately corresponds to the point at which the measured stress–strain curves in^20^ attain a horizontal tangent). This fitting exercise yielded, for the two alloys considered (denoted as Ti-5553-1 and Ti-5553-3 in^20^) ${\sigma }_{y}^{\mathrm{min}}=334$ MPa and ${\sigma }_{y}^{\mathrm{min}}=312$ MPa, respectively. The authors also measured S–N curves for these two alloys (at relatively low stress ratio, *R* = 0.1) and found that both alloys displayed a fatigue endurance limit, measured as 330 MPa and 310 MPa for Ti-5553-1 and Ti-5553-3, respectively. These are remarkably close to the values of ${\sigma }_{y}^{\mathrm{min}}$ extracted from the uniaxial stress–strain curves.

It is well known that fatigue is a macroscopic phenomenon initiated by microscopic plasticity at relatively low levels of stress, and due to local deformation and fracture mechanisms supported and enhanced by cyclic loading. Although the mechanisms are quite complex, and surely they involve complex loss of cohesion, it is logical to think that if the applied stress levels are low enough to not initiate noticeable microscopic plasticity, then the material will not fail by fatigue at such applied stresses. The data, in this case, support this notion; however more complex modelling techniques (accounting for material strain hardening, loss of cohesion, more sophisticated bounds) and a more extensive experimental campaign on different materials would be needed to substantiate this hypothesis.

**Figure 12:**
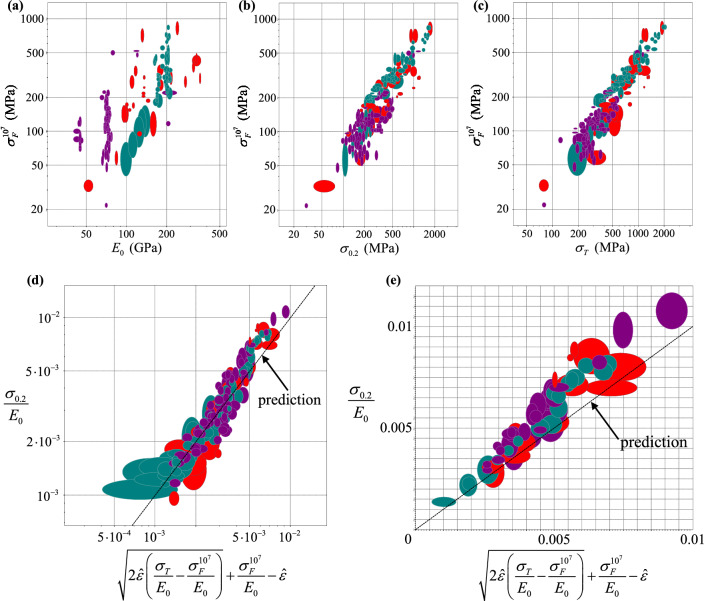
Correlations of the relevant measured mechanical properties for 186 metallic alloys listed in ^21^. The axes of the ellipses shown indicate the measured variability in the quantities plotted. Green, purple and red ellipses indicate ferrous alloys, lightweight alloys (based on Ti, Al, Mg, Be) and non-ferrous alloys, respectively.

However, to further support the possibility of a link between the fatigue endurance limit of metals and their quasi-static monotonic response, we analyse data from a material database^21^, as shown in Fig. 12. The database stores mechanical properties for a large number of engineering materials. In particular, for unreinforced metallic alloys, it reports (i) the yield stress ${\sigma }_{0.2}$, intended as the stress at a plastic strain of 0.2%, (ii) the tensile strength ${\sigma }_{T}$, intended as the peak stress in uniaxial tension, (iii) the Young’s modulus ${E}_{0}$, and (iv) the fatigue strength at 10^7^ cycles ${\sigma }_{F}^{{10}^{7}}$, which is in practice considered as the fatigue endurance limit. Figure 12a–c show the correlation between such fatigue strength and elastic modulus, yield strength and tensile strength, respectively, for the 186 materials for which the four properties above are measured via mechanical test (rather than estimated, by approaches similar to that in^22^). The fatigue strength in general increases when these three parameters increase, and in first approximation it does so according to power-laws of exponents close to 1.

We now assume that the stress versus strain response of metallic alloys can be described by Eq. (14), implying a negligible heterogeneity in elastic properties, moderate variability of the yield stress and negligible strain hardening modulus; the proof stress ${\sigma }_{\widehat{\varepsilon }}$ at a certain plastic strain $\widehat{\varepsilon }$ (in this case ${\sigma }_{0.2}$, evaluated at plastic strain $\widehat{\varepsilon }=0.002$) can be determined by finding the intersection of Eq. (14) with the line of equation $\bar{\sigma}={E}_{0}(\overline{\varepsilon }-\widehat{\varepsilon })$. Under our hypothesis, we assume that ${\sigma }_{F}^{{10}^{7}}={\sigma }_{y}^{\mathrm{min}}$. With regards to ${\sigma }_{y}^{\mathrm{max}}$, we assume that for materials with small strain hardening modulus it is ${\sigma }_{T}\approx {\sigma }_{y}^{max}$; the logic of this is that for a material with a locally perfectly-plastic response, global plastic collapse (and consequent ultimate failure) will have occurred when the applied stress attains the yield stress of the strongest SVE. This leads to the equation

28 $$\frac{{\sigma }_{0.2}}{{E}_{0}}=\sqrt{2\widehat{\varepsilon }\left(\frac{{\sigma }_{T}}{{E}_{0}}-\frac{{\sigma }_{F}^{{10}^{7}}}{{E}_{0}}\right)}+\frac{{\sigma }_{F}^{{10}^{7}}}{{E}_{0}}-\widehat{\varepsilon }.$$

Equation (28) makes mathematical sense for ${\sigma }_{T}\ge {\sigma }_{F}^{{10}^{7}}$, which is satisfied for all materials in the dataset investigated. To further ensure that it can be solved for ${\sigma }_{F}^{{10}^{7}}$, it must also satisfy $\left({\sigma }_{T}-{\sigma }_{0.2}\right)/{E}_{0}\ge \widehat{\varepsilon }/2$. In Fig. 12d we plot measured data for a subset of 149 metallic alloys satisfying the condition above. It is clear that the quantities at the two sides of Eq. (28) are correlated. In Fig. 12e we present the same information as in Fig. 12d, but imposing the further condition ${\sigma }_{T}<1.2{\sigma }_{0.2}$, to exclude materials with very pronounced strain hardening. The correlation between the two sides of Eq. (28), including this time only 60 materials, is plotted in linear scale (rather than logarithmic), to highlight the discrepancies between data and prediction (included in both Fig. 12d,e). Again, Eq. (28) provides a good description of the measured data.

In consideration of the enormous impact that correlations similar to Eq. (28) would have in engineering practice, we think that the preliminary findings highlighted in this section should be reinforced by more extensive investigations, and we hope that some of the readers will join us in this.

## Concluding remarks

Monte Carlo analyses and FE simulations were used to study the effects of microscopic variations of elastic modulus, yield strength and hardening modulus upon the macroscopic elastic–plastic response of model heterogeneous materials. Volume elements consisting of regular arrays of SVEs were generated and their uniaxial and multiaxial stress–strain responses were predicted. The mechanical properties of the SVEs were taken as linear elastic, followed by incompressible J2 plasticity with constant strain hardening modulus and isotropic hardening. Mechanical properties were different in each SVE, according to uncorrelated random fields of uniform probability density. Microscopic variations of either the elastic modulus, the yield stress or the hardening modulus were studied, and their macroscopic effects were investigated. The main conclusions and observations of the study are as follows:

- The macroscopic elastic modulus and the yield stress extracted from experiments are lower than the spatial averages of their microscopic counterparts.
- Heterogeneous materials with a local perfectly-plastic response may display an apparent macroscopic strain-hardening.
- The macroscopic response of a heterogeneous solid is scarcely sensitive to the shape of the probability density function governing the microscopic spatial variability of the mechanical properties, but depends strongly on the relative variance of such probability density.
- The onset of yield in a heterogeneous material occurs at stresses much lower than the proof stress; the higher the degree of heterogeneity, the lower is the macroscopic stress to cause the first local yield.
- Microscopic variation of stiffness or yield stress induce tension/compression asymmetry, strain localisation by shear banding and necking instability in uniaxial tension.
- The strain at which tensile necking occurs in ductile materials increases with the degree of heterogeneity.
- Heterogeneity in plastic properties results in an inverse size effect as well as in a pronounced dependence of the response on the shape of the specimen.
- An elastically heterogeneous material, comprising an array of plastically incompressible domains with isotropic hardening, displays a pressure-sensitive macroscopic response with non-isotropic hardening.
- A preliminary investigation on the application of the findings of this paper suggests that the fatigue endurance of solids might be estimated from a detailed analysis of the stress versus strain curves measured in quasi-static monotonic experiments.
